# The Gut–Brain Axis in the Neuropsychological Disease Model of Obesity: A Classical Movie Revised by the Emerging Director “Microbiome”

**DOI:** 10.3390/nu11010156

**Published:** 2019-01-12

**Authors:** Elena Niccolai, Federico Boem, Edda Russo, Amedeo Amedei

**Affiliations:** 1 Department of Experimental and Clinical Medicine, University of Florence, Largo Brambilla 3, 50134 Florence, Italy; federico.boem@gmail.com (F.B.); edda.russo@unifi.it (E.R.); 2 Department of Biomedicine, Azienda Ospedaliera Universitaria Careggi (AOUC), Largo Brambilla 3, 50134 Florence, Italy

**Keywords:** obesity, microbiota, gut–brain axis, neurological disorders, nervous system, inflammation

## Abstract

The worldwide epidemic of obesity has become an important public health issue, with serious psychological and social consequences. Obesity is a multifactorial disorder in which various elements (genetic, host, and environment), play a definite role, even if none of them satisfactorily explains its etiology. A number of neurological comorbidities, such as anxiety and depression, charges the global obesity burden, and evidence suggests the hypothesis that the brain could be the seat of the initial malfunction leading to obesity. The gut microbiome plays an important role in energy homeostasis regulating energy harvesting, fat deposition, as well as feeding behavior and appetite. Dietary patterns, like the Western diet, are known to be a major cause of the obesity epidemic, probably promoting a dysbiotic drift in the gut microbiota. Moreover, the existence of a “gut–brain axis” suggests a role for microbiome on hosts’ behavior according to different modalities, including interaction through the nervous system, and mutual crosstalk with the immune and the endocrine systems. In the perspective of obesity as a real neuropsychological disease and in light of the discussed considerations, this review focuses on the microbiome role as an emerging director in the development of obesity.

## 1. Introduction

Obesity is an abnormal or excessive fat mass accumulation that affects the health status. The worldwide epidemic of obesity has become an important public health issue, with serious psychological and social consequences since, worldwide, over 650 million adults and 340 million children and adolescents are obese [1]. Obese phenotypes can be associated with some genetic predispositions [2,3] and with sedentary lifestyle. However, these factors alone fail to accurately describe and explain the complexity of the phenomenon. Obesity is a multifactorial disorder which is a result of the interaction of host and environmental factors and its prevalence in high income and upper middle-income countries is more than double that of low and lower middle income countries [4]. Because of that, it constitutes a social problem, especially in those upper middle-income countries, not just affecting the welfare state, but also creating issues in terms of social relations and acceptance, and personal development. Moreover, obesity is considered, sometimes erroneously, as the consequence of an unbalanced and/or mistaken feeding conduct. On the contrary, recent long-term studies reveal a more complex scenario, including neuropsychological and neurobiological factors [5], that in turn involve not only a different categorization of the pathology itself but also suggest that obesity cannot be adequately treated through simple nutritional plans, associated with training and exercise [6]. By considering the complex nature of the pathology (from genetic factors to behavioral and social ones) and given the poor effectiveness of many pharmacological and nutritional approaches, some researchers suggest that “behavioral dimension” should not be neglected, in order to develop new approaches, both preventive and therapeutic, that include obesity within neuropsychological syndromes [5].

In the last years, increasing evidence suggests that microbiome can have a strong impact on hosts’ behavior by different modalities, including interaction through the nervous system, mutual crosstalk with the immune and the endocrine systems, and finally with direct synthesis and managing of neuro-chemicals [7,8,9,10]. In detail, it is well known that the microbiome plays a crucial role in the “gut–brain axis” homeostasis [11], affecting this bidirectional neurohumoral communication, through the production of neuroactive molecules and regulating the circulating levels of some cytokines. Studies have demonstrated that some bacterial strains and their metabolites might target the brain directly, via vagal stimulation, or indirectly through immune-neuroendocrine mechanisms, triggering pro-inflammatory cytokines production and causing neuroinflammation [12]. Given the role of gut microbiome also in energy homeostasis [13,14], it is critical to connect, in a more consistent scenario, the findings relative to metabolic disorders with those concerning neurological and neuropsychological diseases, in the light of general microbiome activities. For instance, dietary patterns, like Western diet, are known to be a major cause of the obesity epidemic, probably promoting a dysbiotic drift in the gut microbiota (GM) [15]. In fact, obese subjects often show neurological comorbidities, such as deficits in memory, learning and executive functions, anxiety and depression, and some evidences support the hypothesis that the brain could be the seat of the initial malfunction leading to obesity [11]. Moreover, by increasing systemic inflammation, microglial activation and affecting vagal nerve activity, the microbiome can indirectly influence hypothalamic gene expression and promote overeating [12]. Given that microbiome acts and connects different host regions, being involved in many physiological and pathological conditions, this review will focus on GM role as emerging director in the obesity pathogenesis and related neurological conditions. As data source, we used Google Scholar and PubMed databases for English-language published material (from 1 January 2008 to 30 August 2018) and examined review and original research articles, using a combination of keywords such as microbiota, microbiome, obesity, neurologic diseases, nervous system, cognitive impairment, gut brain axis, and inflammation. We used our judgment to select articles, sum evidences, and interpret results.

## 2. Why a Neuropsychological View of Obesity?

Obesity occurs when energy intake exceeds energy expenditure over time, and, traditionally, it is considered as the consequence of a sedentary lifestyle and the usual excessive food consumption. Excessive adiposity is a major risk factor for cardiovascular disease, cancer, type 2 diabetes, and mood-related disorders, with obese individuals often suffering social stigmatization [16,17]. Given its multifactoriality, the obesity is a complex disease in which both genetic and environmental factors play a role in its development. Anyway, none of them satisfactorily explains the etiology yet and many details of this pathological condition remain murky. Since differences in the brain could be both a consequence of, and/or an explanatory factor for obesity, recent attention has shifted towards its neurobiological features, in particular in the pathogenic processes and in the clinical-related neurological conditions.

### 2.1. Neurobiological Aspects of Obesity’s Pathogenesis

Feeding, appetite, and energy expenditure are under the control of the central nervous system (CNS), which receives various peripheral signals of energy status and its availability, such as gut hormones and adipokines (signaling molecules released by the adipose tissue) [18,19]. In particular, the hypothalamus regulates the homeostatic feeding behavior, while other neural brain regions, such as insular cortex, orbitofrontal cortex, nucleus accumbens, amygdala, and dopaminergic ventral tegmental area, present groups of neurons implicated in the reward-related non-homeostatic control of feeding [20]. 

Besides the role of orexigenic hormones, like ghrelin [21], and neuropeptides, such as agouti-related protein or neuropeptide Y (activated in hypothalamic neurons during fasting) [22], the food intake is mainly regulated by the energy need from brain, based on its adenosintriphosphate (ATP) disposability [23]. This is possible thanks to the brain regulation of systemic-metabolic pathways. Indeed, an hyperinsulinemic-hypoglycemic state, caused by energy-deficiency, induces the activation of ATP-sensitive K^+^ channels (KATP channels) in the hypothalamus, leading to the increase in hunger feelings, to the gluconeogenesis stimulation, the activation of stress system, and the reduction of pancreatic insulin release [24,23]. First, dysfunctions in any part of this metabolic pathway, such as congenital leptin deficiency, can result in a persistent state of positive energy balance and the obesity development [25,26]. Moreover, it has also been suggested that an alteration in glucose allocation to the brain, keeps the hypothalamic appetite centers activated, engendering a persistent stimulus to food intake, thus causing obesity over time [27,28]. The fact that the brain energy content negatively correlates with BMI (body mass index), supports the viewpoint that the brain can regulate body mass by changing the intake of foods, eventually crucially contributing to the obesity pathogenesis [29]. In addition, the observation that cerebral ATP and phosphocreatine (PCr) levels predict the amount of calories subsequently consumed [30], supports this interpretation. Interestingly, recent studies seem to suggest that alterations in cerebral energy homeostasis may lead to enhanced food intake behavior and, in the long term, to obesity itself [31]. Other investigations have observed, in obese subjects, chronic neuro-energetic deficiency, and lack of satiety perception [32].

In addition to the homeostatic metabolic systems, food intake is regulated by the reward-related mechanisms, through the mesolimbic dopamine (DA) pathways [33], and by stress axis activity [34]. The stress response occurs through the HPA (hypothalamic-pituitary-adrenal) axis [35] activation that ends with the cortisol secretion.

The mesolimbic pathway is a dopaminergic pathway in the brain that connects the ventral tegmental area in the midbrain, to the ventral striatum of the basal ganglia in the forebrain. The DA release plays a role in the pleasure perception and any dysregulation of this process can be linked to the development and maintenance of an addiction [36]. In general, palatable energy-dense foods are often consumed even after energy requirements have been met, and the immoderate consumption of tasty hypercaloric food can lead to a profound state of reward hyposensitivity that is similar to that of drug abuse, leading to the development of compulsive-like eating behaviors [36]. Because of this mechanism, the wide availability of tasty junk food is considered as the major environmental risk factor for obesity [37]. Moreover, stress, triggering DA release [38,39], renders people susceptible to substances that, via reward processing, motivate individuals to overconsumption [40]. At the same time, eating highly pleasant foods further affects reward processing to augment the stress-eating cycle [41,42]. In fact, through negative reinforcement [34], the so-called “comfort food” provides relief from a stressful states, and the hedonic withdrawal that occurs because of the long-term exposure to cortisol [43], is then ingrained as reward-driven habits [44]. Hence, persistent period of stress, that chronically activate the dopaminergic reward system, leads to the development of addiction-like behavior and the beginning of a vicious circle [6] and any decrease in dopamine concentration results in intake of comfort foods, which, in the long time, can drive the weight gain. Finally, among its various functions, cortisol triggers processes that lead to weight gain [45], promoting obesity essentially in two ways. In primis, it is able to increase eating [46,47], by reducing the brain’s sensitivity to leptin [41], regulating neuropeptide Y (NPY) stimulation [48], or strengthening reward pathways [39]. Second, it promotes fat deposition, especially in the abdominal region [49,50] (of note: abdominal obesity represents a marker for longterm cortisol levels) [51]. Indeed, chronic stress enhances food intake, engendering an HPA axis’ hyper activation that leads to the obesity evolution. Hence, obesity can be in part explained by the dysregulation of fundamental neurobiological mechanisms, which lead to irregular eating behaviors, and the interconnection between metabolic disturbances and psychological aspects must be still elucidated.

### 2.2. Obesity and Neurological Comorbidities

Clinical studies indicate an association between obesity and neurological disorders, concerning both the central and peripheral nervous system (Table 1).

**Figure 1:**
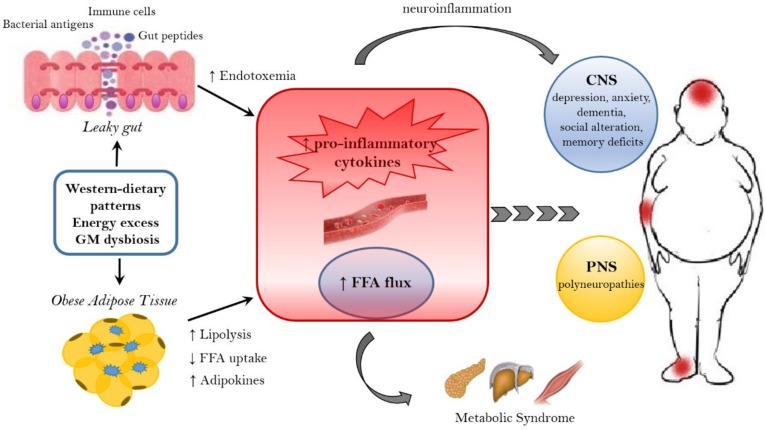
Mechanisms linking obesity to neurological comorbidities. Western-dietary patterns, rich in saturated fat and simple sugars, excessive food intake, and gut microbiota (GM) dysbiosis are related to obesity and its neurological comorbidities through the establishment of an inflammatory state. A dysbiotic microbiota contributes to the leaky gut syndrome, allowing the translocation of gut peptides and bacterial products that increase the peripheral inflammatory tone inducing neuroinflammation. In addition, the dysfunctional obese adipose tissue lead to the increased circulation of inflammatory cytokines, adipokines and FFA. FFA, beside the action on peripheral tissue, where they contribute to the establishment of a metabolic syndrome, have a detrimental effect on both the CNS and PNS. In the CNS, neuroinflammation and lipotoxic FFA can lead to dementia, cognitive impairment, anxiety, and depression, whereas in the PNS the end result are peripheral neuropathies. FFA = free fatty acids. CNS = Central nervous system. PNS = peripheral nervous system.

The net energy overload observed in obese patients, leads to the increase in the adipose mass, characterized by adipocytes hypertrophy and/or hyperplasia, which is necessary for the storage of surplus calories. The expansion of the adipose tissue contributes to its dysregulation that includes an altered adipokine secretion profile, the activation of resident macrophages and the establishment a low grade metabolic inflammation (Figure 1).

In particular, adipose tissue alteration is characterized by the production of: pro-inflammatory cytokines interleukin-1Beta (IL-1Beta), IL-6, tumor necrosis factor-alfa (TNF-alfa), monocyte chemotactic protein 1 (MCP-1) [52]; inflammatory mediators C-reactive protein and leptin [53] and increased release of free fatty acids (FFA) [54]. The circulating FFA exert a lipotoxic effect on peripheral and nervous tissues, being responsible for the establishment of a metabolic syndrome and the onset of neurological diseases [55,56]. In detail, FFA can target both the CNS and the peripheral nervous system (PNS), leading to cognitive impairment, other CNS diseases and peripheral neuropathies [56,57,58]. Epidemiological and prospective studies show that obesity increases the risk of mild cognitive impairment [59,60,61,62,63], Alzheimer’s disease and dementia [64,65]. Moreover, a high BMI is associated with episodic memory deficits [66], altered decision making [67], attention-deficit/hyperactivity disorder [68,69,70], learning and memory problems [71] both in adults and adolescents [72,73]. In obese patients, the establishment of a low grade inflammation and the altered FFA metabolism seems to provoke structural changes in the brain, such as the reduction of hippocampal volume [74] and the atrophy of frontal, temporal and subcortical regions [75], as well as impaired executive functions [76,77,78]. In response to the increase of dietary fat, inflammation and metabolic dysfunctions affect the brain, in particular the hippocampus, an essential structure for learning and memory, and susceptibility to aging-related atrophy [78,79]. For example, FFA and triglycerides can directly alter hippocampus functions [78], and circulating adipokines provoke an alteration of the blood–brain barrier (BBB) permeability [79]. The latest mechanism establishes a feed-forward cycle of injury, since the unruly entrance of pro-inflammatory cytokines and chemokines, immune cells [80] and FFA into the hippocampus, engenders neuroinflammation, promoting neurodegeneration [81,82]. Moreover, the insulin-resistance associated with a high fat (HF) diet affects microvascular perfusion in the hippocampus, decreasing cognitive function in rodents [83]. In addition, the hypothalamus, which is not protected by the BBB, is particularly vulnerable to circulating lipotoxic fats and adipokines [84]. The hypothalamic inflammation occurs early in response to a HF diet feeding and induces microglial and astrocyte reactivity [84] with the gliosis promotion and the propagation of pro-inflammatory signaling [85]. Finally, the HF diet leads to the apoptosis of hypothalamic neurons [86] linked to the pathogenesis of Alzheimer’s disease. Additionally, metabolic disturbances in obesity affecting the HPA axis regulation, can further worsen depression [87], a condition commonly associated with obesity [88,89].

The pathophysiological consequence of obesity also affects the PNS. First, the autonomic ganglion and postganglionic fibers are not protected by BBB and susceptible to obesity-mediated inflammation [90]; second, sensory neurons and peripheral sensory receptors lie out of the blood–nerve barrier, being exposed to metabolic insults [91]. Recent evidence suggests that obesity is a risk factor for the development of autonomic neuropathy and polyneuropathy, a distal-to-proximal loss of sensory perception [92,93,94,95]. This can be due to the chronic dysfunction secondary to obesity and lipid-induced inflammation and it is characterized by the accumulation of macrophages and the expression enhancement of the pro-inflammatory cytokines in peripheral nerves [96,97]. Interestingly, various findings, obtained in human beings and in animal models, show that dietary intervention, bariatric surgical procedures and the resolution of obesity improve polyneuropathy, decreasing neuropathic pain [98], and ameliorate the cognitive performances [99,100,101,102].

Despite all these studies, the fine mechanisms linking obesity to cognitive and neurological disorders still need to be elucidated. Even if inflammation plays a prominent role in the development of neurological dysfunction, additional research should be focusing on the identification of new pathways linked to obesity-related inflammation, such as the gut–brain axis communication.

## 3. The Microbiota–Gut–Brain Axis

The intestine and the brain are intimately connected by the gut–brain axis, a complex bidirectional system in which the central and enteric nervous system communicate involving endocrine, immune and neuronal pathways. Communication and functions of this axis are regulated by the GM at the point that the concept of microbiota–gut–brain (MGB) axis has been introduced to underline the pivotal role of GM in the development of metabolic and neurological diseases.

The microbiota represents the community of microbes (bacteria, archaea, viruses, and fungi) that reside in a particular habitat (e.g., the gut microbiota) and establish with the host a mutually beneficial relationship (while the “microbiome” represents the collective genomes of microorganisms) [103]. In particular, the microbiota offers benefits to the host maintaining the gut integrity [104], harvesting energy [105], providing protection against pathogens [106] and regulating the immune system [107]. The human gastrointestinal tract holds more than 1000 bacterial species, mainly located within distal ileum and colon, which belong prevalently from *Bacteroidetes* and *Firmicutes* phyla. The GM com-position is highly dynamic and susceptible to rapid changes in response to external factors as diet, stress, smoking, infections, or perturbation of the healthy state [108]. In turn, changes in GM com-position and function, named dysbiosis, can be responsible for the development of various diseases (e.g., colorectal cancer) [109,110], and, can contribute to the disruption of the molecular dialogue between gut and brain [111].

The MGB axis is composed of the CNS, the autonomous nervous system (ANS), the neurons of the enteric nervous system (ENS), the HPA-axis and the GM. In particular, signals from the brain influence the motor, sensory, and secretory modalities of the gastro-intestinal tract, regulate the inflammatory process and influence the GM structure [112]. In turn, visceral messages from the gastro-intestinal trait can influence brain function [112,113]. For instance, under stress conditions, the cortisol released following HPA axis activation alters the gut permeability and barrier function, thus affecting the GM composition [112].

Conversely, the gut microbiome influences the brain functions modulating the levels of various brain transmitters (i.e., serotonin) [114] and circulating cytokines, that can exceed the BBB [115,116].

Growing evidence, involving studies in germ-free (GF) animal models, which intestinal flora is missing from birth, and humans exposed to probiotic agents or antibiotics, suggests that several pathologic conditions may be affected by a MGB axis dysregulation, such as autism spectrum disorders [117,118,119], anxiety/depression [120], and obesity [121,122,123].

### 3.1. Microbiome and Energy Harvest

The intestinal microbiome plays a key role in digestion and absorption of nutrients, regulating energy homeostasis through different mechanisms as energy extraction from food and the modulation of fat storage by the short chain fatty acids (SCFAs) and monosaccharides absorption.

The first information about the role of bacterial flora in the obesity physiopathology was obtained in GF mouse models [124,125,126]. These mice were significantly leaner than controls, despite introducing more calories from food [127]. Moreover, GF mice showed modified plasma fatty metabolic markers and lower amount of leptin and ghrelin, suggesting an energy imbalance [128]. When GF mice were transplanted with gut bacterial flora obtained from conventionally raised mice, they showed an increase in insulin resistance and in body fat without an observed increment in nutrient intake [123]. This evidence has placed the gut microbiome at the center of a completely new research field, concerning the pathophysiology of obesity. Biochemical and metagenomics data analysis suggested that the “obese microbiota” [14] was able to harvest more energy from the diet and this ability was also transferable. Thus, the colonization of GF mice with an obese microbiota (human or murine) induced an increase in total body fat higher than that one obtained through colonization with a lean microbiota [129]. In addition, when eutrophic GF mice received fecal microbiota from obese women, metabolic complications associated to obesity have been observed [130]. This evidence indicates how rapid, transmissible and flexible can be the relation between food and commensal microorganisms in obesity and metabolic syndrome.

Animal obesity models and obese humans present a similar microbial phylum taxonomic rank dysbiosis. In particular, humans and mice share two main phylum: *Firmicutes* and *Bacteroidetes*; an alteration of *Firmicutes/Bacteroidetes* ratio was observed in several obesity studies in both mice [131] and humans [14]. Indeed, the *Firmicutes* could break down indigestible carbohydrates and converting them into absorbable energy products [132,133,134]. However, in a meta-analysis study the observed alteration in the ratio *Firmicutes*/*Bacteroidetes* seemed to be unrelated to weight differences [135]. Also, the reduction in microbial diversity and alteration of particular microbial families or species have been observed in obesity conditions [114], such as the increase of *Proteobacteria* [136].

The complex interplay between host genetics, gut microbiome and environmental factors is crucial for the obesity pathophysiology (for instance, monozygotic twins showed a more similar GM profile than did dizygotic twins) [137]. Dietary pattern could also affect the bacterial structure, for example, westernized diets increase the abundance of *Clostridia* (*Firmicutes* phylum) populations that could extract more energy from the diet, consenting higher energy utilization [138]. The unused extra energy is then accumulated as fat deposits.

While different processes, by which an ‘‘obese microbiota’’ can affect body weight balance [127,139,140] have been indicated, the increased energy harvest via colonic fermentation and SCFAs’ production is the most direct [14]. Bacterial enzymes (specific glycoside hydrolases) metabolize otherwise not digested by humans food components, like vegetable fibers (such as resistant starch, cellulose, and inulin) that cannot be metabolized by human enzymes. The final product of this process are energy-rich substrates, such as SCFAs [141]. SCFAs can provide ≤10% of total daily caloric intake [142]. Obese individuals show significantly increased levels of SCFAs such as acetate, propionate, and butyrate [133,143]. Most of the bacterial SCFAs (in particular butyrate) are derived from the fermentation process of *Clostridia* cluster [144]. SCFAs not only operate as energy substrates for host tissues and bacteria but also act as signaling molecules in the host metabolism, showing a relevant role in mediation of gut motility, regulation of fat storage and appetite [105]. Indeed, dysbiosis induced by the type of diet has been correlated with an acetate increase that promotes hyperinsulinemia [145]. Furthermore, SCFAs can influence other obesity-associated conditions such as insulin resistance and hyperglycaemia [146].

Among the SCFAs, the propionate can be utilized locally through conversion into glucose by intestinal gluconeogenesis or diffuse into the portal vein to be utilized as a substrate for hepatic gluconeogenesis, preventing high SCFAs concentrations in blood [147]. In addition, propionate decreases human lipogenesis and serum cholesterol (in hepatic and non hepatic tissues) [148], and also reduce the fasting blood glucose and hepatic cholesterol in obese rats [149].

In addition, several studies shows acetate benefits on metabolism. Acetate could bind to the receptor GPR43 in several target organs. In adipose tissue, the GPR43 activation inhibits insulin signaling and suppresses fat accumulation, while systemically, it improves insulin sensitivity [150]. Mice deficient in the acetate receptor GPR43 become obese when fed a normal diet, whereas mice who overexpress GPR43 remain lean even when fed an obesogenic diet [150]. In the liver, acetate reduces lipid accumulation and improves liver function and mitochondrial efficiency. In adipose tissue, acetate inhibits fat breakdown but induces the browning of white adipose tissue and metabolic improvements, leading to a reduction in body fat [151]. Finally, prebiotics such as inulin, increased acetate production that crosses the blood–brain barrier of rats and results in reduced grehlin production and so, inducing a decrease of body weight, food intake, and fat mass [152]. Moreover prebiotic fructooligosaccharides increase acetate production, reducing body weight and fat mass because it favors a lower food intake in mice [153].

Microbial metabolites can also regulate the composition of bile acid species. A reduced bile acid amount in the intestine has been associated with inflammation and microbiota overgrowth [154]. Some intestinal bacteria are able to extract energy from the metabolization of bile acids, inducing the activation of bile acid receptors farnesoid X receptor (FXR) and Takeda G-protein-coupled receptor 5 (TGR5). These two receptors maintain insulin sensitivity and glucose tolerance in both liver and intestine [29,30]. In addition, other mechanisms have been proposed to account for the increased microbiota capacity to extract energy from the diet intake [155]:

1. GM influences energy homeostasis by regulating gene expression via complex mechanisms started by SCFAs and monosaccharides [156]. In particular, the commensal microorganisms stimulate monosaccharide cellular uptake [157] and induce lipogenesis by activating the transcription factors carbohydrate response element binding protein (ChREBP) and sterol response element binding protein (SREBP) [155]. Triacylglycerols, produced trought hepatic lipogenesis, are thus sent from the liver to the blood in the form of very low-density lipoprotein and chylomicrons.
2. HF diet triggers an increased absorption of bacterial lipopolysaccharide (LPS) (an endotoxin in the cell wall of Gram-negative bacteria) from the gut lumen to the bloodstream inducing low-grade inflammation, by activating B cells or dendritic cells activating and cytokine production [158]. The inflammation could also be stimulated by endotoxemia condition [157]; moreover, also the damaged gut barrier might contribute to this metabolic endotoxaemia [159].
3. GM induces a suppression of angiopoietin-like protein 4 (ANGPTL4) in the intestinal epithelium. ANGPTL4 is a circulating enzyme produced by liver, gut and adipose tissue that inhibits LPL; its suppression provokes an increase of triacylglycerol storage in the adipose tissue [127].

Of note, relevant evidence suggests that both the consumption of fermentable carbohydrates and the supplementation of SCFAs result in positive effects on host physiology and energy homeostasis. The resistant starch (RS) is a fermentable dietary fiber used as a carbohydrate source in food. Obanda and colleagues used obesity-prone and obesity-resistant rats to examine how weight gain and fat accretion relate to fermentation levels and GM after feeding RS [160]. Obese-prone rats fed with RS at 20% of the weight of the diet did not gain more body fat than the same type of rats fed the same diet except without the RS [160]. It could be possible that fermentation of prebiotics increased energy expenditure but with contemporary greater energy absorption, and so without net gain in weight and body fat. The authors hypothesized that dietary RS decreases body fat accumulation through stimulating endogenous GLP-1 and PYY production [161]. However, the majority of these recent researches have investigated the effect of SCFAs on animal models or in particular tissue or metabolic process. Since SCFAs have different and parallel metabolic processes that affect energy homeostasis, more studies are needed to bring these effects together in order to elucidate the real impact of SCFAs [139].

### 3.2. Microbiome and the Brain

As previously reported, the gut microbiome affects the host’s CNS functions (as cognitive and vegetative activities) through the MGB axis. CNS functions, vice versa, may influence the structure of the microbiota that inhabits the intestinal lumen [112]. Several studies suggest that this mutual interplay has a pivotal role in the occurrence of metabolic disorders, such as diabetes and obesity [162], but also in the development of eating and stress-related neuropsychiatric disorders, including [163] anxiety and depression.

**Figure 2:**
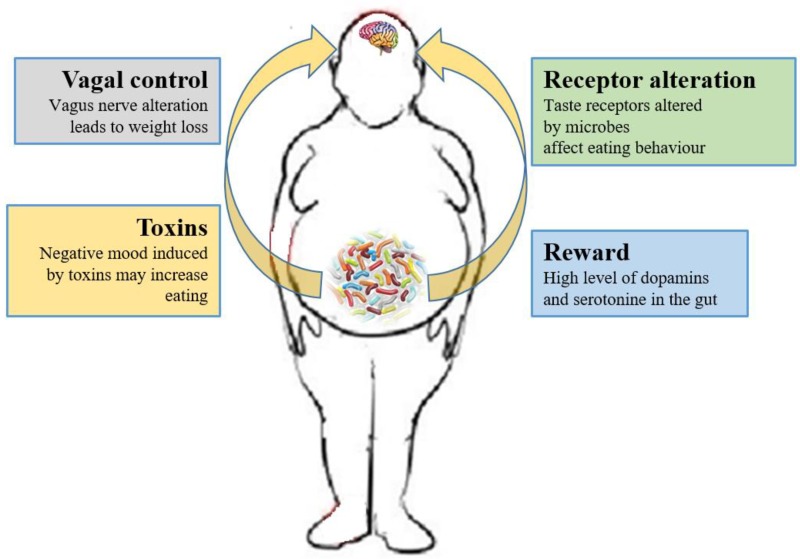
Relations between gut microbiota and eating behavior. The gut microbiota controls the eating behavior by several mechanisms, including changes to receptors such as taste receptors, regulation of reward pathways, production of toxins that alter mood, and deviating neurotransmission via the vagus nerve.

Currently, researchers are focusing on whether the microbiome can have effects on the CNS process and on the hedonic and homeostatic control of dietary intake [164] (Figure 2).

As previously described, the SCFAs produced by the microbiota have systemic effects, but they can also directly signal to the (enteral and central) nervous system, via stimulation of the vagus nerve, or indirectly through immune-neuroendocrine processes [158].

The role of the vagus nerve is important in the MGB axis because it connects the 100 million neurons of the enteric nervous system to the “nucleus tractus solitaries” [112]. The information inserted in this communication axis is then delivered to the hypothalamus, which modulates energy balance, appetite and dietary intake [20]. This information also includes signals from commensal microorganisms, linking the cognitive and emotional nucleus of the CNS with peripheral gut activity, finally leading to host eating control. Experiments showed that transection or blockade of the vagus nerve could induce a dramatic weight loss [165]. On the other hand, vagus nerve functions appear to drive extreme eating behavior in satiated animals when they are treated with norepinephrine [166]. The parasympathetic vagal activity was linked with weight loss also in anorexia nervosa [167], indicating that vagal signaling, involved in the modulation of body weight, can lead to pathological anorexia, and other CNS disorders as anxiety-depressive behaviors and autism [168,169].

The vagus nerve could be stimulated by enteroendocrine cell hormones as gut peptide YY (PYY) and glucagon-like peptide 1 (GLP-1). The satiety hormone PYY inhibits gut motility, increases gut transit time, and reduces appetite [170], while GLP1 decreases appetite and improves insulin sensitivity [171]. The bacterial SCFAs could alter the release of those hormones into systemic circulation binding to their specific enteroendocrine G-protein coupled receptors (GPRs) [172,173]. Through the GPRs activation, SCFAs induce leptin expression, which produces the suppression of the appetite and GLP-1 production [174]. The increased plasma GLP-1 and PYY levels inhibit ghrelin secretion [174] and regulate appetite by releasing it into the blood stream [175]. Acetate, the main SFCA produced by the microbiome, has a direct effect in the suppressing of appetite via central hypothalamic process [152]. The increased acetate production, due to an altered gut microbiome, induces the stimulation of the parasympathetic nervous system with improved secretion of ghrelin, obesity and hyperphagia [145]. Lactate, another bacterial metabolite produced by *Enterobacteriaceae*, *Lactobacilli* and *Bifidobacteria*, is the favorite substrate for neuronal cells and could prolong the postprandial satiety [176].

Furthermore, the microbiome can affect the central control of appetite by producing neuroactive metabolites as tryptophan, serotonin, gamma-aminobutyric acid, endocannabinoid ligands, and ghrelin. These bacterial metabolites are the exact analogs of the mammalian hormones implicated in behavior and mood signaling [177]. More than half of the dopamine and the majority of the body’s serotonin are produced at gut level [178]. Indeed, components of bacterial flora, as *Bacillus cereus*, *Escherichia coli* [179], *B. subtilis*, *B. mycoides*, *Serratia marcescens*, *Proteus vulgaris,* and *Staphylococcus aureus* [180] can produce dopamine. The probiotic *B. infantis* 35624 improves blood levels of tryptophan [181], a precursor of serotonin that mediates appetite-suppressant function by the regulation of melanocorting neurons, which control body weight homeostasis [182,183]. Moreover, the lactic acid producing bacteria could secrete the neurochemicals histamine [184] and GABA [185] that is involved in the regulation of feeding and energy balance [186,187]. Interestingly, GABA stimulates the same neuroreceptors that are targeted by anti-anxiety drugs (benzodiazepines).

A few cross-sectional studies have demonstrated the association of mood alteration and anxiety with microbiota dysbiosis [188] due to the alteration of the tryptophan metabolism [189]. Of note, some probiotics with anti-depressive actions also showed anti-obesity effects, confirming that gut bacteria modulation could be beneficial also for obesity-related depression [190]. The administration of *Lactobacillus helveticus* reestablished the altered *Firmicutes/Bacteroidetes* ratio in mice with HF diet with the contemporary reduction of anxiety-like behaviors [191]. In addition, *Bifidobacterium* and *Lactobacillus* decreased depressive-like behaviors in association with reduced IL-6 and TNF-α level in blood [192]. These bacterial strains could also decrease the inflammatory tone, affecting both mood disorders and obesity. The microbiome-mediated inflammatory processes, associated with obesity, affect the CNS, leading to important changes in several neurocircuities, including neuroendocrine activity (impaired feedback response to cortisol) neurogenesis (impaired in the hippocampus) and neurotransmitter metabolism (alteration in dopamine system and basal ganglia) [168]. Moreover, the changes in feeding behavior could be attributable to dysphoria induced by the microbiome. One potential mechanism by which dysphoria can influence eating involves bacterial virulence gene expression and host pain perception [193]. The production of virulence toxins often is triggered by a low concentration of growth-limiting nutrients. Detection of simple sugars regulates virulence and growth of several gut bacteria [194]. Microbiota could injure the gut epithelium when certain nutrients are absent, manipulating behaviors through pain signaling [195,196,197] Moreover, pain perception (nociception) requires the presence of an intestinal microbiota in mice [198], while fasting has been shown to increase nociception by a vagal nerve mechanism [199]. In addition, some bacteria could stimulate the endocannabinoid system, which affects gut barrier activity, host metabolism [200] and the homoeostatic and hedonic control of appetite and food intake [201]. Brain reward signaling is mediated by the dopaminergic mesolimbic system involved in the pathogenesis of obesity [202]. Increased colonic propionate reduces anticipatory reward responses to high-energy foods via striatal pathways [203].

Microbiome is involved also in social behavioral alterations due to diet. An animal model study reports that maternal high-fat diet (MHFD) induces a shift in microbial ecology that negatively affects offspring social behavior, showing a linki between MHFD, intestinal microbiota disequilibrium, ventral tegmental area plasticity and social behavior variations in descendants [204]. Fewer oxytocin immunoreactive neurons in offspring hypothalamus were also observed. The administration of *Lactobacillus reuteri* to the descendants was able to correct the oxytocin concentrations and social deficits progeny.

Moreover, studies linked microbiome to the regulation of satiety and taste. One study found that the absence of a bacterial flora in GF mice, considerably diminished expression of gut satiety peptides. GF mice also demonstrated also impaired taste receptors for fat on gut and tongues resulting in an augmented calorie intake from fats [128]. In addition, enteroendocrine cells expressed different taste receptors (bitter, sweet, fat, and umami) and their activation induced secretion of GLP-1, CKK, and ghrelin [205] In another study, GF mice preferred more sweets and showed greater numbers of sweet taste receptors when matched to normal mice [206]. Furthermore, the oral administration of *L. acidophilus* NCFM increased gut expression of opioid and cannabinoid receptors in epithelial cell culture [207]. These findings indicate that microbiome could also affect food preferences by the modification of receptor expression or transduction [207]. Interestingly, changes in taste receptor expression and activity were observed after gastric bypass surgery. This procedure also induces also gut microbiome alteration leading to satiety and food preferences changes. Several studies also indicate a connection between cravings and the GM profile. For example, subjects who are “chocolate desiring” showed different microbial metabolites than “chocolate indifferent” people, despite identical food intake [208]. Finally, obesity, microbiome, and diet may also affect episodic and semantic memory [15]. Recent studies documented beneficial effects of probiotics on cognitive functions in humans [209]. On the contrary, in cohort of brain fogginess (BF) patients (consuming probiotics) with unexplained abdominal bloating, gas, pain and distension (possibly caused by SIBO—small intestinal bacterial overgrowth) and D-lactic acidosis, the discontinuation of probiotics along with a course of antibiotics led to symptoms’ resolution [210]. Probably, probiotic use may increase the accumulation of bacteria in the small intestine, resulting in disorienting brain fogginess as well as belly bloating.

### 3.3. The Role of Microbiome-Driven Inflammation

As widely discussed over the review, the immune system plays a crucial role in the gut–brain axis communications since immune mediators are important messengers of this complex dialogue and, consequently, it mechanistically links the function’s impairments in both brain and gut, as shown by the association between chronic gut inflammation and psychological morbidity [211,212,213,214]. The immune system plays a key role in obesity and correlated pathologies, such as in the colorectal cancer [215]. In obese patients, a chronic low-grade inflammatory state is maintained [216,217] and the peripheral inflammation, with the activation of innate immune components (like TLRs) and the loss of intestinal barrier integrity, can lead to neuro-inflammation. Interestingly, recent studies have demonstrated that dysbiosis and inflammation may concur to the development of various diseases, including obesity and depression disorders [218]. In addition, numerous studies have now clearly confirmed that the gut microbiome can, qualitatively and quantitatively, shape the host immune responses, both in the gut and in systemic tissues. In this way, the GM influences the concentration and profile of cytokines present in any given individual and, in turn, differentially affects the brain function. For example, GF mice show numerous immune abnormalities, including impaired antibody responses, diminished numbers of T and B lymphocytes and a defective production of cytokines (such as IL-10, TNF-alfa, IL-6 and IL-1) [219,220]. Moreover, selective GM constituents shape specific aspects of adaptive and innate immunity, including the differentiation of particular effector T-cell lineages [221,222,223]. The obesity-associated dysbiosis is characterized by a remarkable inflammatory potential of microbiota [224,225], which is able to activate innate and adaptive immunity in the gut and beyond, increasing the inflammatory tone by TLRs activation and production of pro-inflammatory cytokines [226]. Sen and colleagues have demonstrated that a dysbiotic microbiota (high sugar diet-associated) alters the vagal gut–brain communication [208], producing an inflammatory state that increases gut permeability. The result is the passage of LPS and pro-inflammatory cytokines from the lumen to the lamina propria (triggering an inflammatory response) and so, microglia activation in the nodose ganglion and finally leading to vagal remodeling [224]. Moreover, a microbiota with enhanced pro-inflammatory activity has been demonstrated to be able to promote intestinal inflammation, inducing colitis and metabolic syndrome [14,226]. The loss of intestinal barrier integrity, seems to be a crucial step in the obesity pathogenesis and related diseases, including neurological disorders [219,227] (Figure 1). In fact, the leaky gut allows the translocation of Gram-negative bacteria’s components into the mesenteric lymph nodes and the circulation, boosting the release of pro-inflammatory cytokines (especially TNF-alpha), via TLR2/4 direct or indirect activation [227,228,229,230], and increasing the production of IgA and IgM [231,232]. In general the gut permeability can be considered the direct consequence of the dysbiotic microbiota-driven local gastrointestinal inflammation [233,234], and notably, in obese mice, the prebiotics’ supplementation can improve the gut integrity, reducing the weight gain [235]. The leaky gut and the associated-inflammation lead to peripheral insulin resistance and hyperglycemia, supporting the obesity establishment; moreover, the increased inflammatory cytokines in the peripheral system can affect the BBB integrity, contributing to the development of mood disorders [234]. Bruce-Keller and colleagues have linked obesity, microbiome, and neurologic dysfunction, demonstrating the ability of HF diet-dysbiotic microbiota to increase inflammatory gene expression in the medial prefrontal cortex associated with anxiety and memory impairment [236]. Moreover, the inflammation generated by HF diet-dysbiotic microbiota can activate the microglia [237], a process observed in various neurological disorders [237,238,239,240] and associated with weight gain and bacteria-driven hyperphagia [241].

Finally, expanding the knowledge of the mechanisms underlying the triggers of such inflammatory responses in obesity could offer unique opportunities for intervention strategies reducing the risk of related neurological conditions and supporting personalized treatments [242].

## 4. Conclusions and Future Directions

All these data suggest the presence of a deep link between the composition of the microbiota and the development of obesity. This not only in light of the known impact of microbiome activities on metabolic pathways and their modification, but also because the well described comorbidities, obesity-associated (e.g., anxiety and depression), show a possible association with the activities of the microbiome itself. This suggests several hypotheses. On the one hand, the presence of the microbiota in all these conditions could mean that the microbiota itself is involved as a linking factor between neuropsychological and metabolic disorders. Alternatively, the microbiota could be directly associated with obesity and, given its impact on the nervous system, presenting a more relevant causal role in the development of the comorbidities themselves. Still differently, the emergence of some neuropsychological disorders could be either generated or enhanced by the microbiota modulation and thus its functions, in relation to obesity. Finally, the most radical hypothesis is that the microbiome itself, due to its functional pervasiveness, may constitute a possible causal trigger of both obesity and its comorbidities, which should therefore be reviewed and redefined in another light. In all these scenarios, which will have to be investigated experimentally as soon as possible, it appears that the microbiome plays a central role. Although its role is not yet fully described from a general point of view, its impact on several levels and on multiple systems is now undeniable. In other words, from these studies, it clearly emerges that microbiota is a sort of director, that from behind the scenes (compared to other biological phenomena much more investigated) connects (or perhaps even directs) elements and components of the host organism according to a more integrated, systemic perspective.

Such a systemic perspective should be intended as a methodological attitude rather than an intrusion, performed by different experts, into other areas of investigation. As a matter of fact, by looking at obesity as a neuropsychological disorder, does not mean to diminish the importance of classical factors and relative approaches in the study of this important disorder. Rather, it means embracing a more global vision of the disease (given its complexity and multifactoriality) that is, precisely, systemic. Consequently, shifting attention to the role played by the microbiome, given its pervasiveness and capillarity of interactions, can be a new and proficuous way to investigate the relationships of that system and opening a new prospective for preventive and therapeutic approaches of obesity.

Finally, future studies are needed to deep the knowledge on the mechanisms underlying the MGB signaling and to identify the molecular pathways that could be modulated by microbiota-based strategies. In this way, we can have new therapeutic approaches to avoid obesity and its comorbidities. We strongly believe that additional translational and functional studies in this light will be useful to improve targets and approaches to treat obesity and its neurological comorbidities. 

## Figures and Tables

**Table 1:** Clinical studies showing an association between obesity and central nervous or peripheral neurological diseases.

Study	Sample Size	Patients’ Features	Findings	Ref.
**Central nervous system disorders**				
Holtkamp et al., 2004	97 men	Children with attention-deficit/hyperactivity disorder (ADHD)	Obesity development independent of ADHD diagnosis	[69]
Elias et al., 2005	551 men, 872 women	Individuals with healthy body weight, overweight, obese	In men, obesity association with adverse cognitive effects	[59]
Cournot et al., 2006	1660 men, 1576 women	Healthy workers (32–62 years old)	Higher BMI association with lower cognitive scoresand higher cognitive decline	[60]
Boeka et al., 2008	20 men, 48 women	Caucasian and African American extremely obese patients	Evidence of specific cognitive dysfunction in extremely obese individuals	[73]
Sabia et al., 2009	3788 men, 1343 women	White individuals	Multiple effects of obesity on cognition	[61]
Hassing et al., 2010	140 men, 277 women	Swedish twin registry	Midlife overweight association to lower overall cognitive function in old age	[62]
Anstey et al., 2011	71529 individuals	Participants evaluated for any type of dementia	Overweight and obesity in midlife increase dementia risk	[64]
Dahl et al., 2013	280 men, 377 women	Swedish adoption and twin study of ageing	Midlife overweight or obesity responsible of lower cognitive function and cognitive decline in late life	[63]
Yau et al., 2014	30 obese, 30 lean adolescents	Obese without insulin resistance or metabolic syndrome	Uncomplicated obesity may result in subtle brain alterations	[72]
Cheke et al., 2016	14 men, 36 women	8 Obese individuals, 16 overweight, 26 lean	Higher BMI association with lower performance on the what-where-when memory task	[66]
Navas et al., 2016	35 men, 44 women	38 Normal weight, 21 overweight, 20 obese	Obesity is linked to a propensity to make risky decisions	[67]
Kummer et al., 2016	92 patients and 19 controls	Children and adolescents: autism spectrum disorder (ASD) andADHD	Higher risk of overweight and obesity in ASD and ADHD	[70]
Peripheral nervous system diseases				
Ylitalo et al., 2011	2514 adults aged ≥ 40 years	Individuals with peripheral neuropathy,peripheral vascular disease (PVD), alower-extremity diseases (LEDs).	Obesity and cardiometabolic clustering markedly increased the likelihood of LEDs	[92]
Tesfaye et al., 2005	1172 patients	Patients with type 1 diabetes mellitus.	Higher BMI independently associated with the incidence of neuropathy.	[93]
Ziegler et al., 2008	195 patients and 198 controls	Population-based MONICA/KORA Augsburg Surveys aged 25–74 years.	Waist circumference association with peripheral arterial disease (PAD)	[94]
Singleton et al., 2014	21 obese, 51 lean controls	Non-diabetic obese patients referred for Roux en Y bariatric surgery compared with lean controls.	Asymptomatic neuropathy is common in very obese patients independent of glucose control	[95]
